# 2′-Fucosyllactose Ameliorates Inflammatory Bowel Disease by Modulating Gut Microbiota and Promoting MUC2 Expression

**DOI:** 10.3389/fnut.2022.822020

**Published:** 2022-02-17

**Authors:** Qianqian Yao, Linlin Fan, Nan Zheng, Christophe Blecker, Véronique Delcenserie, Huiying Li, Jiaqi Wang

**Affiliations:** ^1^State Key Laboratory of Animal Nutrition, Institute of Animal Sciences, Chinese Academy of Agricultural Sciences, Beijing, China; ^2^Gembloux Agro-Bio Tech, University of Liège, Gembloux, Belgium; ^3^Department of Food Science, Faculty of Veterinary Medicine, University of Liège, Liège, Belgium

**Keywords:** 2′-fucosyllactose, colitis, gut microbiota, anti-inflammation, MUC2

## Abstract

Gut microbiota dysbiosis, together with goblet cells dysfunction has been observed in ulcerative colitis cases. This study aims to evaluate the potential of 2′-fucosyllactose (2′-FL) supplementation in inhibiting intestinal inflammation through regulating gut microbiota, protecting goblet cells, and stimulating mucin secretion. 2′-FL was orally administered to C57BL/6J mice daily (400 mg/kg bw) for 21 days and 5% dextran sulfate sodium (DSS) was used to induce the colitis in the last 7 days. Meanwhile, fecal microbiota transplantation (FMT) was conducted to test the roles of gut microbiota in the remission of colitis by 2′-FL. Gut microbiota alteration was analyzed through 16S ribosomal RNA (16S rRNA) sequencing. Periodic acid-Schiff (PAS), immunofluorescence staining, as well as mucin 2 (MUC2) and NOD-like receptor family pyrin domain containing 6 (NLRP6) messenger RNA (mRNA) expression in colon fragments was performed and detected. The results showed that the DSS + 2′-FL mice were found to have a slower rate of weight loss, lower disease activity index (DAI) scores, and longer colon lengths than the DSS group (*p* < 0.05), so in the FMT recipient mice which received fecal microbiota from the DSS + 2′-FL group. In addition, the data revealed that 2′-FL relieved the disorder of DSS-induced gut microbiota, including decreasing the high abundance of mucin-utilizing bacteria in the DSS group, such as *Bacteroides, Lachnospiraceae NK4A136, Lachnospiraceae*, and *Bacteroides vulgatus*. PAS and immunofluorescence staining showed that 2′-FL treatment promoted the recovery of goblet cells and enhanced MUC2 and NLRP6 expression, which was also observed in the FM (DSS + 2′-FL) group. Moreover, NLRP6, which has been proved to be a negative regulator for Toll-like receptor 4/myeloid differential protein-8/nuclear factor-kappa B (TLR4/MyD88/NF-κB) pathway, was upregulated by 2′-FL in colon tissue. In conclusion, this study suggests that 2′-FL ameliorates colitis in a gut microbiota-dependent manner. The underlying protective mechanism associates with the recovery of goblet cells number and improves MUC2 secretion through TLR4-related pathway.

## Graphical Abstract

2′-fucosyllactose (2′-FL) acts as a suppressor of inflammation in DSS-induced colitis, by which regulating gut microbiota composition, improving the recovery of goblet cells and MUC2 secretion, and subsequently affect the TLR4-related inflammatory pathway. The work suggests 2′-FL as a promising functional food supplement for intestinal health. 
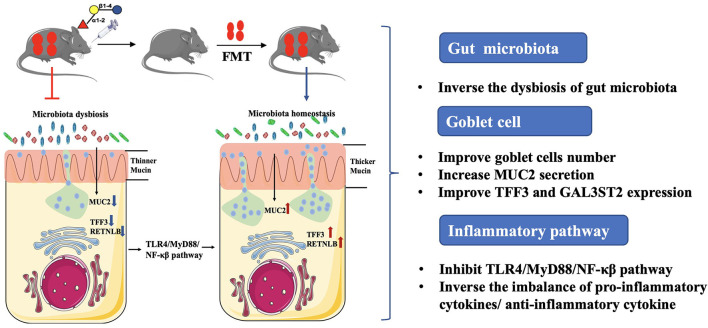

## Introduction

Inflammatory bowel disease (IBD), a chronic, recurring inflammatory response, is rising in prevalence across the world and diagnosed with increasing frequency during adolescence and early adulthood (1). Epidemiological and clinical evidence suggest that it results from aberrant crosstalk among environmental factors, gut microbiota, and the immune system (2, 3). Although the mechanism of IBD onset is still unclear, it is widely accepted that dysbiosis of intestinal flora is closely associated with its development (4, 5). Considering the flexible manipulation of intestinal flora (6, 7), their potential reshaping through providing specific substrates, such as food-derived nutrients, may provide a novel approach for the prevention of IBD.

Human milk is a nutritious source of proteins, fats, micronutrients, prebiotics, and probiotics. As the initial food for neonates, it contributes significantly to their growth during the first month. Although breast milk and milk powder have similar major components, their functions have been proven to be different. The lower frequency of inflammation in breastfed infants, compared within non-breastfed infants, mainly results from a high concentration of diverse structural human milk oligosaccharides (HMOs) in breast milk (8, 9). To date, more than 200 kinds of HMOs have been identified, including fucosylated, sialylated, and neutral non-fucosylated ones (10). Due to the fucosylated and sialylated radicals, HMO cannot be digested by digestive enzymes in the gastrointestinal tract and is, instead, fermented by gut microbiota in the colon (11). 2′-fucosyllactose (2′-FL) is the most abundant HMO in human milk, accounting for about 25% and has been approved to serve as a dietary supplement and nutritional health care product by the US Food and Drug Administration (FDA). 2′-FL has been found to play a crucial role in regulating the immune system of newborns by stimulating the growth of beneficial intestinal bacteria (such as *Bifidobacterium* and *Lactobacillus*) (12). Moreover, it has been reported that 2′-FL would inhibit the adhesion of pathogens to the surface glycans of epithelial cells, thus limiting the virulence of some pathogens (13, 14). Though it has been discovered that 2′-FL could regulate the composition of gut microbiota, it is still unclear whether the gut flora is necessary for 2′-FL to exert physiological function.

The intestinal mucus layer lies at the interface between bacteria and host epithelial cells. Mucin 2 (MUC2), the major component of intestinal mucin, is produced by goblet cells in Toll-like receptors (TLRs)- and NOD-like receptor family pyrin domain containing 6 (NLRP6)-dependent manner (15, 16). MUC2 is glycosylated at Ser/Thr residues with oligosaccharides composed of N-acetylgalactosamine, galactose, sialic acid, N-acetylglucosamine, and fucose (17). In clinical and animal experiments, a thinner mucus layer and lower level of glycosylation of MUC2 have been found in colitis cases (18, 19), which likely contribute to reduce commensal fitness and drive the microbial dysbiosis in colitis. Due to the structural similarity between mucin O-glycans and 2′-FL, we hypothesized that 2′-FL can modulate mucin function *via* the gut microbiota, thus improving the intestine function.

To address the above questions, in this study, the colitis mice model was constructed to evaluate the potential of 2′-FL to prevent inflammation in IBD. Moreover, the responses of gut microbiota to 2′-FL during the anti-inflammatory process, as well as the MUC2 secretion were investigated, thus providing new insights to understand the relationship between HMOs and intestine health.

## Materials and Methods

### Chemicals

Dextran sulfate sodium (DSS) [molecular weight (MW): 36,000–50,000, cat#CD4421] was obtained from Coolaber (Beijing, China). 2′-FL (purity ≥ 98%, cat#GY1141) was purchased from Huich Technology Corporation Ltd. (Shanghai, Beijing). Mouse interleukin-1 beta (IL-1β) (cat#85-BMS6002), interleukin-6 (IL-6) cat#85-BMS603-2), and tumor necrosis factor α (TNF-α) (cat#85-BMS607-3) ELISA kits were purchased from Thermo Fisher Scientific (Waltham, Mississippi, USA), while interleukin-10 (IL-10) (cat#ab255729) and interleukin-17 (IL-17) (cat#ab100702) were purchased from Abcam (Cambridge, England, UK). Ampicillin (cat#A105483-5g), vancomycin (cat#V105495-5g), and neomycin (cat#N109017-5g) were obtained from Aladdin (Shanghai, Beijing, China) and metronidazole (cat#443-48-1) was purchased from Solarbio (Beijing, China).

### Animal Experiments

A total of 18 C57BL/6J male mice (18–20 g, wide type, 6–8 weeks old) were purchased from Vital River Laboratory Animal Technology Corporation Ltd., Beijing, China. The animals were kept in cages at a constant temperature of 25°C and relative humidity of 50% under specific pathogen-free conditions. The mice were acclimated for 7 days before the formal experiment. The protocol applied in this study was approved by the Committee on the Ethics of Animal Experiments of the Chinese Academy of Agricultural Sciences (Beijing, China; permission number: IAS-2021-03), which conforms to internationally accepted principles in the care and use of experimental animals (NRC, 2011). Surgical procedures were performed under anesthesia and all the efforts were made to minimize the suffering of the mice.

### 2′-Fucosyllactose Treatment and DSS-Induced Colitis Model Construction

**Figure 1:**
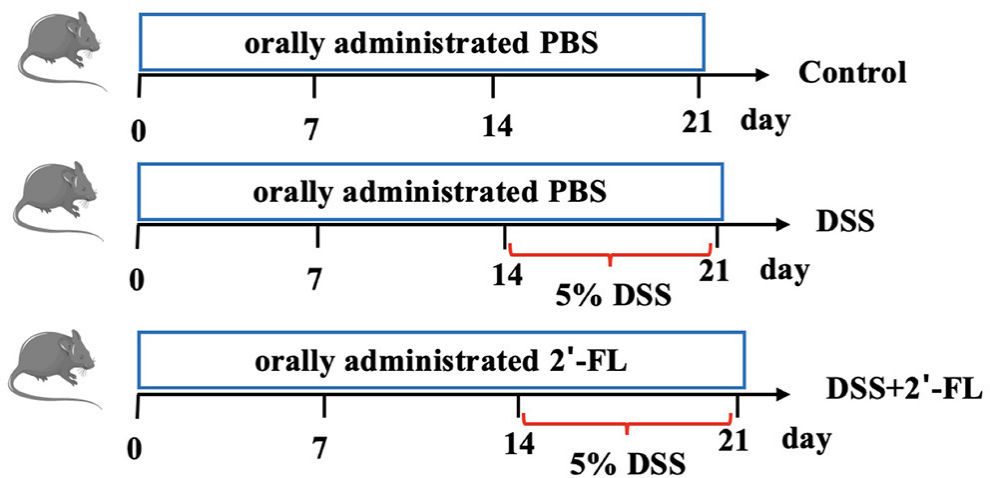
Experimental workflow for evaluating the effects of 2′-fucosyllactose (2′-FL) on colitis.

The mice were randomly assigned into three groups: the control group, the DSS group, and the DSS + 2′-FL group, *n* = 6. During 0–21 days, mice in the DSS + 2′-FL group were orally administrated with 0.3 ml 400 mg/kg bw 2′-FL, once daily, while the mice in the control group and the DSS group received equal volumes of phosphate-buffered saline (PBS). The daily dose of 2′-FL was selected according to previous studies (20) and on the basis of the safe daily intake range per kg body weight in infants, which is equivalent to 20 g one day for an adult (21, 22). Between days 14 and 21, mice in the DSS and DSS + 2′-FL groups were administrated with 5% (w/v) DSS in their drinking water ad libitum for 7 continuous days to construct the colitis model (Figure 1). During the experiment, the disease activity index (DAI), consisting of weight loss (0, none; 1, 0–5%; 2, 5–10%; 3, 10–15%; and 4, >15%), stool features (0, normal stool; 1, soft stool; 2, wet and soft stool; 3, semi-loose and watery stool; and 4, loose and watery stool), and stool bleeding (0, normal stool; 1, obscure and indistinguishable blood in the feces; 2, obscure but distinguishable blood in the feces; 3, semi-loose and watery stool and obvious blood in the feces; and 4, deep and large range of red blood in the feces), was assessed daily according to references (23, 24). Scores were given as a summation of three indexes according to the severity of each parameter. At the end of the experiment, all the mice were killed under anesthesia and blood and colon tissue were collected.

### Fecal Microbiota Transplantation

**Figure 2:**
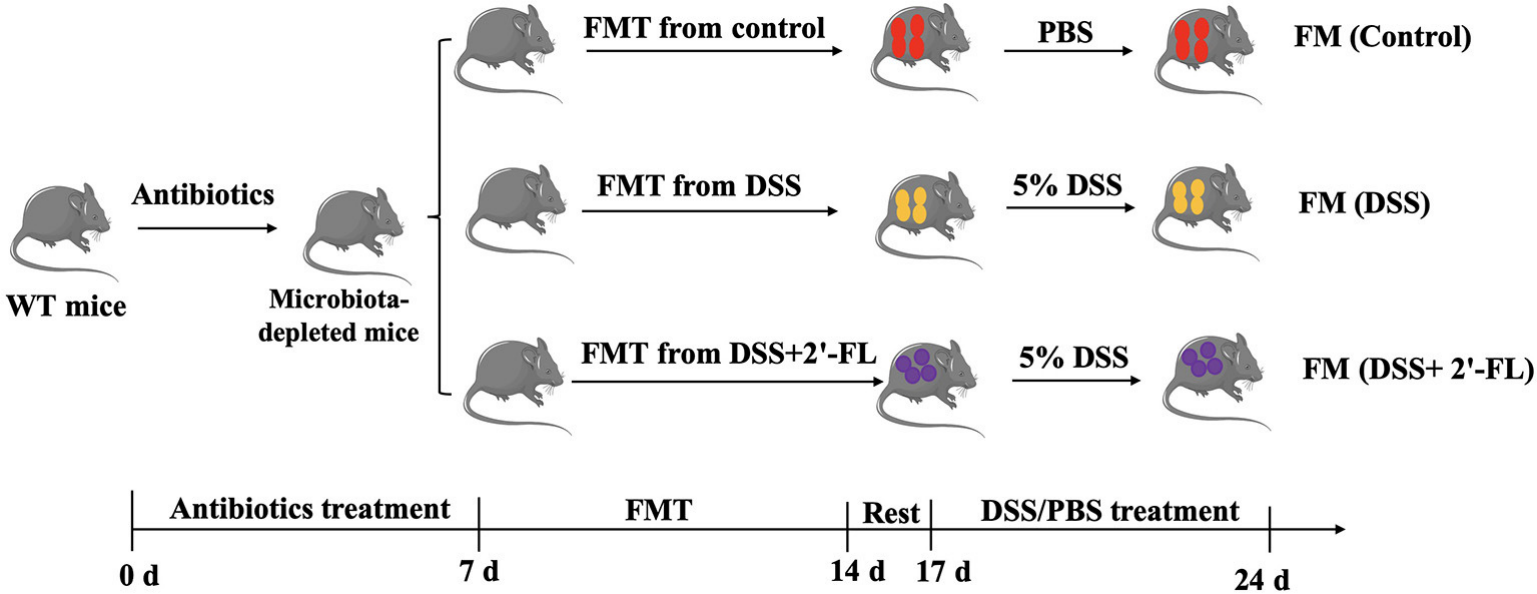
Experimental workflow of fecal microbiota transplantation (FMT).

Fecal microbiota transplantation was performed as described in previous studies (25). The mice were orally administered 0.3 ml antibiotic cocktails (200 mg/kg ampicillin, 200 mg/kg neomycin, 200 mg/kg metronidazole, and 100 mg/kg vancomycin) for 7 consecutive days continuously, once a day, for intestinal flora depletion. Fresh feces samples were collected from donor mice on the 5th day after DSS treatment. The fresh samples were suspended in sterile PBS [1 pellet (with similar weight)/ml] and vortexed for 5 min. Then, the suspension was centrifuged at 800 g for 5 min at 4°C to remove solid particles. The fecal suspension obtained by centrifugation was pooled. The unused fecal suspension is divided and frozen. The bacterial suspension was prepared every 2 days and the recipient mice were intragastrically administered 0.3 ml of the bacterial suspension once a day for 7 days, as shown in Figure 2. All the operations were carried out on a clean bench and all the materials used were sterilized to prevent other bacterial contamination.

### Histopathology

The cleaned distal colon segments were rinsed with precooled PBS three times to remove the content. It was then placed into a 10% paraformaldehyde solution for 24 h, followed by dehydrating, paraffin embedding, sectioning, and staining (H&E, PAS). Finally, histological analysis was performed according to a previously reported method (26).

### Inflammatory Cytokines Detection

Levels of interleukin-1β (IL-1β), IL-6, TNF-α, IL-17, and IL-10 in the serum were detected by ELISA, according to the instructions of the manufacturer.

### Deoxyribonucleic Acid Extraction and 16S Full-Length Sequencing

The fecal DNA was extracted using the Magnetic Soil and Stool DNA Kit (Tiangen Biotechnology Corporation Ltd., Beijing, China), according to the instructions of the manufacturer. The DNA concentration of the samples was measured with the Qubit dsDNA HS Assay Kit and Invitrogen Qubit 3.0 Fluorometer (Thermo Fisher Scientific, Waltham, Massachusetts, USA). The universal primer set 27F: AGRGTTTGATYNTGGCTCAG and 1492R: TASGGHTACCTTGTTASGACTT was used to amplify the full-length 16S rRNA gene from the genomic DNA extracted from each sample. SMRTbell libraries were prepared from the amplified DNA by the SMRTbell Express Template Prep Kit 2.0 and then sequenced on a single PacBio Sequel II 8M cell using the Sequel II Sequencing kit 2.0 (Pacific Biosciences, Menlo Park, California, USA).

The raw reads generated from sequencing were filtered and demultiplexed using SMRT Link software (version 8.0) with the minPasses ≥ 5 and minPredicted Accuracy ≥ 0.9, to obtain the circular consensus sequencing (CCS) reads. The UCHIME algorithm (version 8.1) was used to detect and remove chimera sequences to obtain clean reads. Sequences with similarities ≥ 97% were clustered into the same operational taxonomic unit (OTU) by USEARCH (version 10.0) (27) and the OTUs with abundance <0.005% were filtered (28). Taxonomy annotation of the OTUs was performed based on the naive-Bayes classifier in QIIME2 using the SILVA database (29) with a confidence threshold of 70%. The alpha diversity was calculated and displayed by the QIIME and R software, respectively. Beta diversity was determined using QIIME to evaluate the degree of similarity in microbial communities from different samples. Principal coordinate analysis (PCoA), heat maps, unweighted pair group averaging (UPGMA), and non-metric multidimensional scaling (NMDS) were used to analyze the beta diversity.

### Immunofluorescence Staining

Frozen sections were prepared and blocked with 10% sheep serum at 37°C for 1 h and then were added primary antibodies including anti-MUC2 and anti-NLRP6 overnight at 4°C; the next day, sections were incubated with fluorescent sheep antirat secondary antibody in 0.5% BSA-phosphate buffered saline with Tween (PBST) solution (diluted 1:1,000) for 1 h at room temperature. The nucleus was stained with 4′, 6-diamidino-2-phenylindole (DAPI) dye (diluted with PBS at 1:1,000) at room temperature for 8 min in the dark place. The slices were sealed with antifluorescence quenching solution and observed with a Zeiss Fluorescence Microscope (LSM 800, Germany, UK).

### Ribonucleic Acid Isolation and Quantitative RT-PCR

Colon tissues were collected and grounded in liquid nitrogen. RNA was extracted by the Trizol method. After being added with 75% ethanol, samples were centrifuged at 10,000 g for 10 min and then the supernatant was removed. RNA was purified by the following steps: 30 μl 8 mol/l LiCl and 270 μl RNase-free water were added at −20°C standing for 30 min and then centrifuged at 10,000 g for 10 min. After the precipitate was dissolved by 90 μl Rnase-free water, 10 μl 3 mol/l CH_3_COONa and 200 μl absolute ethyl alcohol were added and incubated for 30 min at −20°C and then centrifuged at 14,000 g for 30 min. After the supernatant was removed, 75% ethanol was added to precipitated RNA and samples were centrifuged at 8,000 g for 10 min. RNA samples were transcribed into complementary DNA (cDNA) (42°C for 10 min, 65°C for 10 s, stored at 4°C) by the PrimeScript™ RT Reagent Kit (Takara Biotechnology Incorporation, Kusatsu, Shiga, Japan). The primers of MUC2 (F: AACACAGTCCTGGTGGAAGG; R: CATTGTCAGGTCCCACACAG); NLRP6 (F: AAGGT GAAGGAGAGGAATG; R: GAAGAGCCGATTGAAAGTG); intestinal trefoil factor (TFF3) (F: CATGTCACCCCCA AGGAGTG; R: AGGTGCATTCTGCTTCCTGC), and resistin-like beta (RETNLB) (F: CACCCAGGAGCTCAGAGATCTAA; R: ACGGCCCCATCCTGTACA) were synthesized and genes expression were detected and glyceraldehyde phosphate dehydrogenase gene (GAPDH) were adopted as housekeeping gene. The results were calculated by 2^−Δ*ΔCt*^ method.

### Western Blot

In total, 0.1 g colon tissue was collected and grounded in liquid nitrogen. 100 μl radio immunoprecipitation assay (RIPA) buffer (containing 50 mM Tris, 1.0% Triton X-100, 0.1% sodium dodecyl sulfate (SDS), 150 mM sodium chloride, 0.5% sodium deoxycholate, and protease and phosphatase inhibitor cocktail) were added and then the tissue was processed by Ultrasonic Cell Pulverizer (JY88-IIN) for 5 min. After fully lysed, it was centrifuged at 12,000 g for 15 min, 1 μl supernatant was taken for quantitative determination using the bicinchoninic acid (BCA) Protein Assay Kit. For rest of the solution, a 200 μl 5 × NuPage lithium dodecyl sulfate (LDS) loading buffer was added. After being boiled, equal amounts of protein from each sample were separated by SDS-PAGE and transferred to a PVDF membrane (Millipore, Billerica, Massachusetts, USA). After being blocked in 5% skim milk dissolved in Tris buffered saline with Tween (TBST), membranes were incubated with primary antibodies for 2 h at 25°C and then probed with secondary antibodies conjugated with horse radish peroxidase (HRP) for 1 h at 25°C. Primary antibodies used in this study were anti-NLRP6 (cat#SAB1302240), anti-Toll-like receptor 4 (TLR4) (cat#bs-1021R), antimyeloid differential protein-88 (MyD88) (cat#bs-1047R), antinuclear factor kappa-B (NF-κB) (cat#51-0500) and anti-β-actin (cat#A01011). All the signals were visualized and analyzed by Image J (1.53a).

### Statistical Analysis

*In-vitro* tests (ELISA test) were performed in three independent experiments (and in triplicate each time) and expressed as mean ± SEM. Animal study results were expressed as mean ± SEM with six biological repeats. All the records were analyzed by ordinary one-way ANOVA with Tukey's analysis to assess differences between the groups. *V*alues of *p* < 0.05 were considered as statistically significant. The GraphPad Prism version 9.0 was applied to drawbar charts of the above data.

## Results

### 2′-FL Significantly Remitted the Colitis Induced by DSS in C57BL/6J Mice

**Figure 3:**
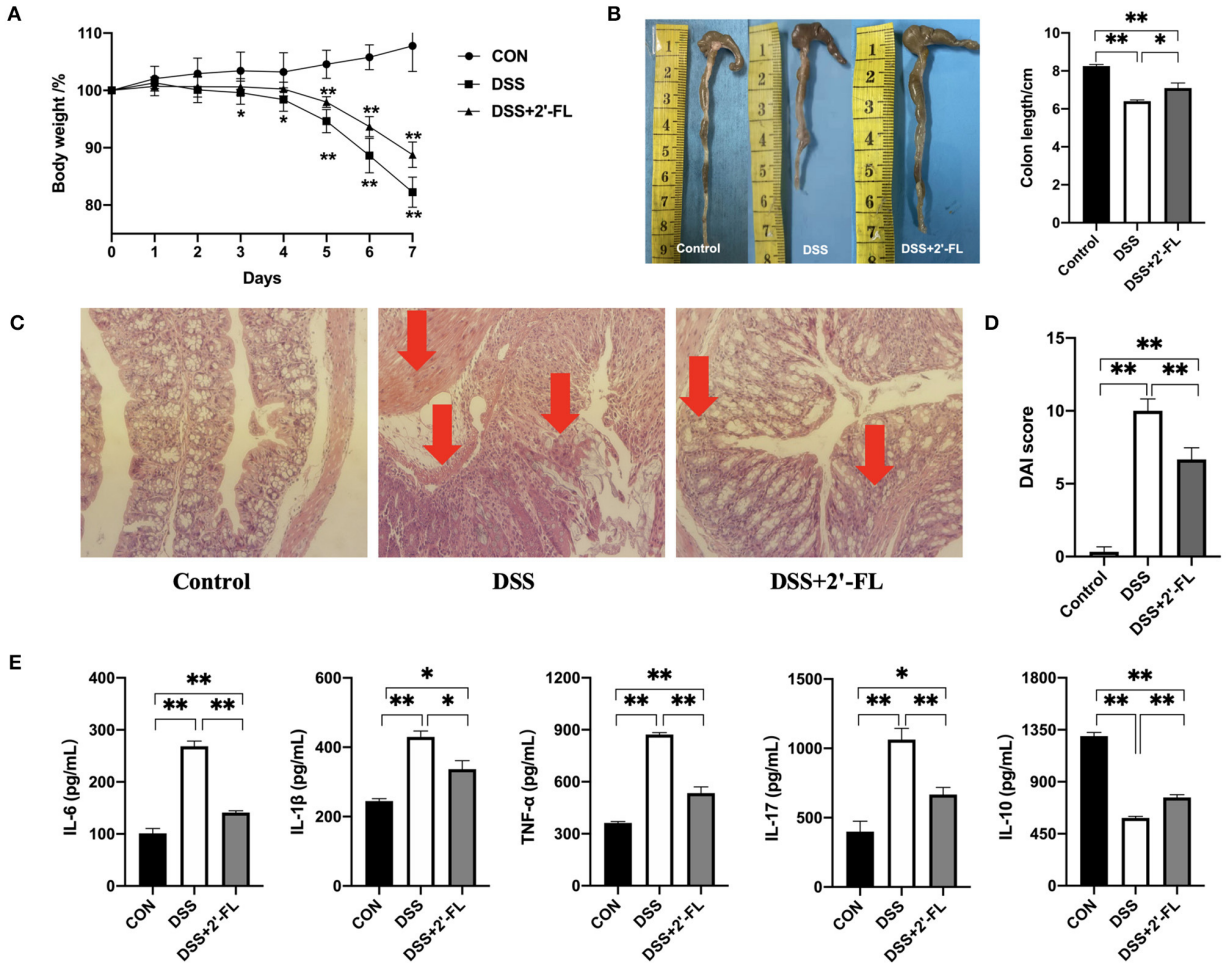
2′-FL remits dextran sulfate sodium (DSS)-induced colon inflammation in C57BL/6J mice. **(A)** Changes in the body weight of mice after receiving 5% DSS water; **(B)** The representative images (left) and statistical analyses (right) of colon length; **(C)** Representative images of colon by H&E staining (100X); **(D)** Disease activity index (DAI) indices; **(E)** Cytokine levels in serum of C57BL/6J mice detected by ELISA. Significance was determined using one-way ANOVA analysis and expressed as mean ± SEM. *p < 0.05; **p < 0.01.

To evaluate the potential of 2′-FL to inhibit inflammation, we adopted the colitis model, a widely acknowledged inflammatory model, in this study. The results showed colitis was successfully induced in the mice after the treatment with DSS for 7 days, as reflected in their significantly decreasing body weight, shorter colon lengths, and higher DAI scores in comparison to those of the control group (*p* < 0.05; Figures 3A,B,D). Moreover, H&E staining showed normal histological architecture and cytology without inflammation were observed in the control group. Mice in the DSS group exhibited severe and diffuse destruction of the epithelial layer and extensive inflammatory cells infiltration in epithelium and lamina propria of the colon (arrows in Figure 3C). In contrast, mice in the DSS + 2′-FL group, which were administered 2′-FL orally for 21 days, showed a less weight loss, lower DAI scores, and longer colon (*p* < 0.05) compared to the DSS group. Additionally, the DSS + 2′-FL group exhibited a lower degree of inflammation in the mucosal and intestinal glands and less inflammatory cells infiltration (arrows in Figure 3C) when compared with the DSS group. The concentrations of proinflammatory cytokines, such as IL-6, IL-1β, TNF-α, and IL-17, were significantly increased in the DSS groups compared with those of the control group (*p* < 0.01), while the level of anti-inflammatory cytokine, IL-10, was remarkably decreased (*p* < 0.01, Figure 3E). Together, these results suggested that 2′-FL effectively relieved the colitis induced by DSS in C57BL/6J mice.

### FMT Verified Gut Microbiota Involvement in 2′-FL's Colitis Mitigating Process

**Figure 4:**
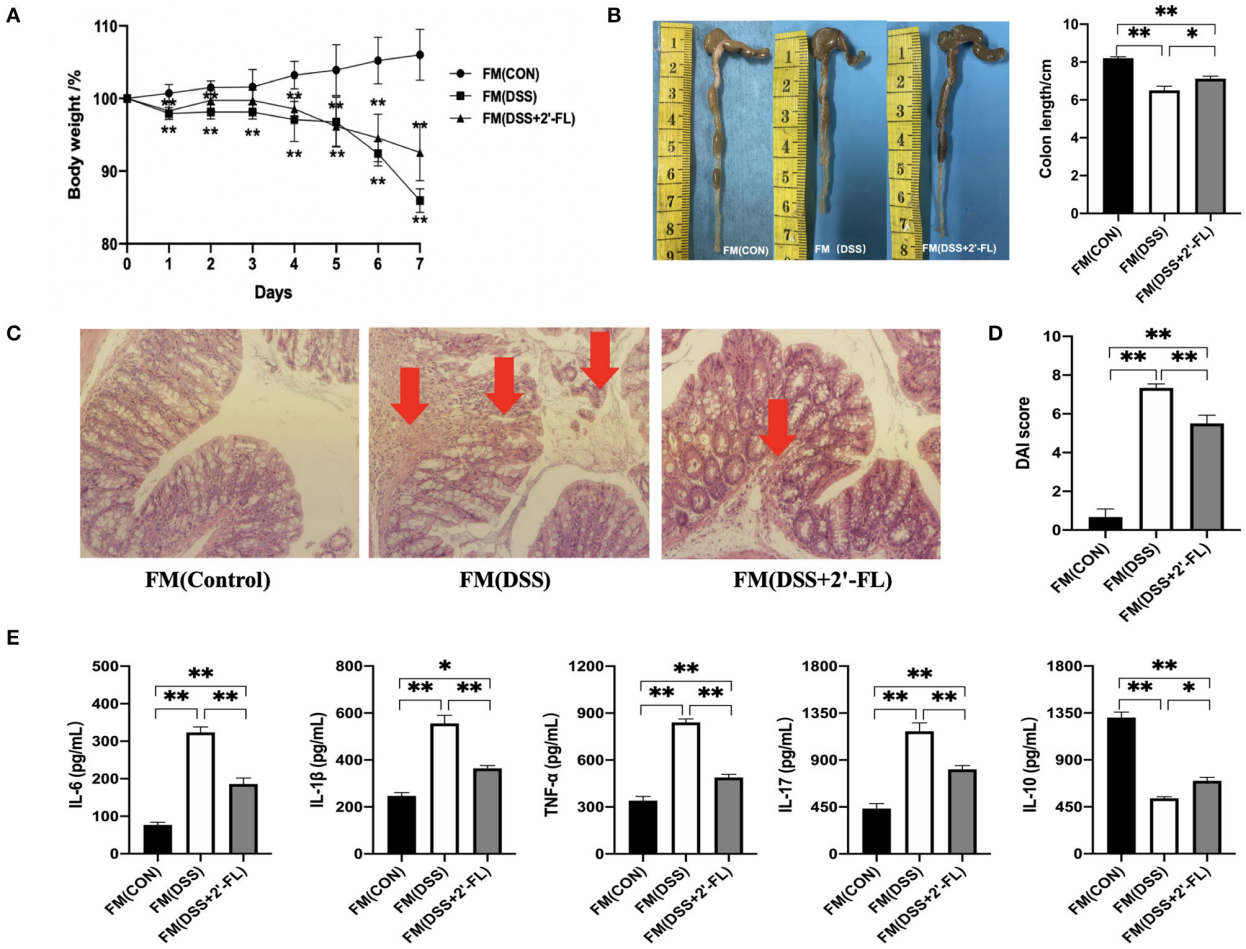
Gut microbiota is involved in the process of 2′-FL mitigation of colon inflammation. **(A)** Changes in body weight of mice after receiving 5% DSS water; **(B)** The representative images (Left) and statistical analysis (Right) of colon length; **(C)** Representative images of colon by H & E staining (100×); **(D)** DAI indices. **(E)** Cytokine levels in serum of FMT C57BL/6J mice detected by ELISA. Significance was determined using one-way ANOVA analysis and expressed as mean ± SEM. *p < 0.05; **p < 0.01.

Gut microbiota are commonly believed to be associated with IBD and influence the functional effects of active substances (30, 31). To investigate whether intestinal flora participated in 2′-FL's mitigation of colitis, wide-type C57BL/6J mice were orally administered an antibiotic cocktail to remove their intestinal microbiota, after which they underwent FMT with fecal matter from the other groups, followed by DSS treatment. Interestingly, the recipient mice in the FMT groups showed similar pathological phenotypes with those of the donor mice, as evidenced by the slower weight loss (Figure 4A), longer colons (Figure 4B), lower DAI scores (Figure 4D), alleviated pathology (Figure 4C), and lower proinflammatory cytokine levels (Figure 4E) in the FM (DSS + 2′-FL) group compared with the FM (DSS) group. These results accumulatively indicated that gut microbiota was necessary for the process in which 2′-FL mitigated colon inflammation.

### 2′-FL Significantly Altered the Composition of Gut Microbiota

**Figure 5:**
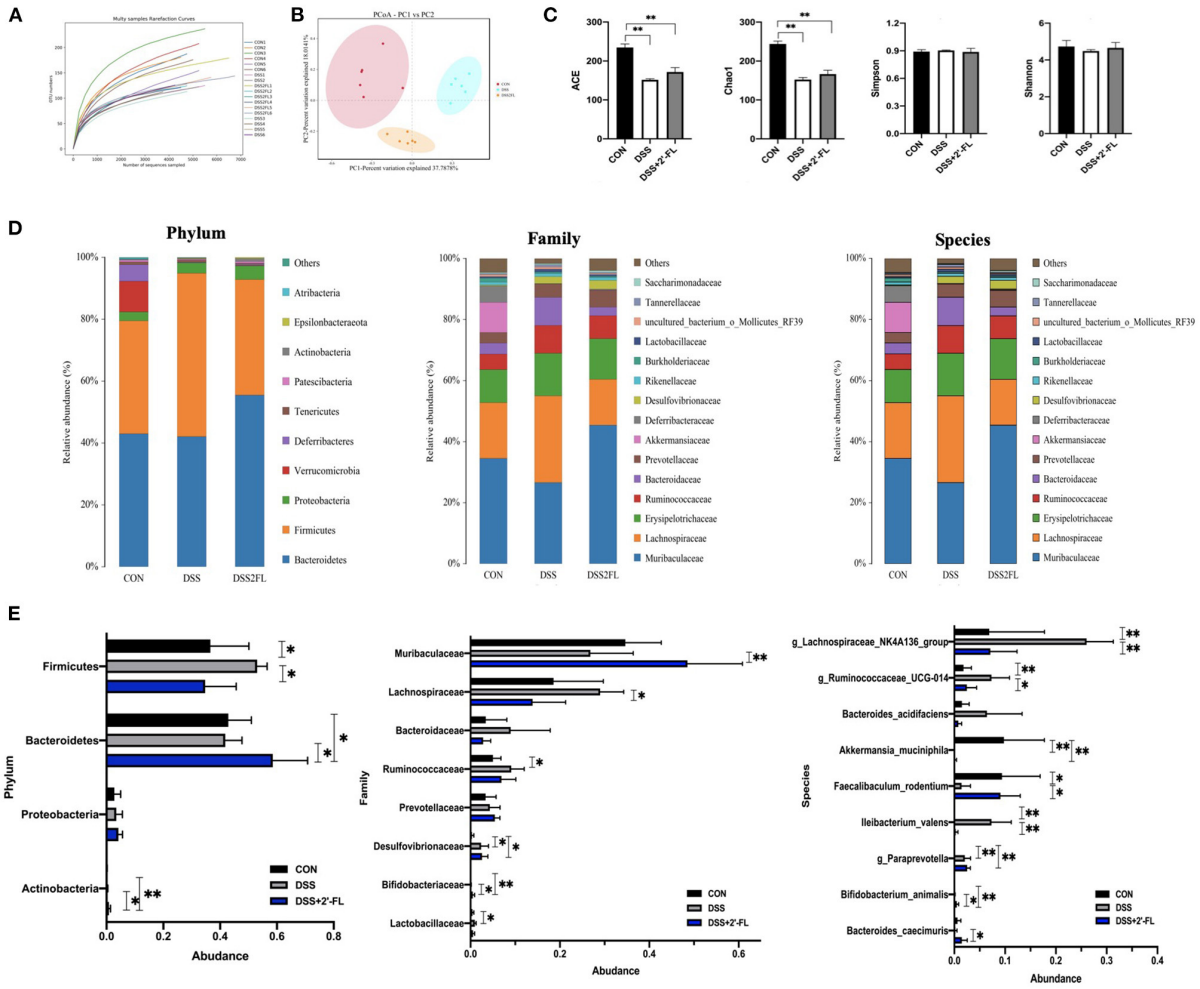
Gut microbiota composition changes in response to the stimuli of colitis and 2′-FL. **(A)** Multi samples rarefaction curve; **(B)** Principal coordinate analysis (PCoA) using the Bray–Curtis metric distances of beta diversity. **(C)** Alpha diversity (ACE, Chao1, Simpson, and Shannon index); **(D)** Stacked bar plots of species distribution at phylum, family, species levels, respectively. **(E)** Representative significantly changed bacteria in phylum, family, species levels across groups. Significance was determined using one-way ANOVA analysis and expressed as mean ± SEM. *p < 0.05; **p < 0.01.

To further ascertain changes in the composition of gut microbiota following treatment with 2′-FL, full-length 16S rRNA sequencing of the fecal microbiota was performed (Figure 5A). As shown in Figure 5B, an apparent cluster separation among the three groups was revealed in the PCoA of the Bray–Curtis distances, indicating that these communities were specific. Alpha diversity was significantly declined (ACE and Chao1) in the DSS group compared with the control group (*p* < 0.05, Figure 5C). The relative abundances of microbes were further analyzed in the groups to determine the species artificially altered by 2′-FL and *p*-values were calculated by ANOVA. At the phylum level, *Firmicutes* was the most predominant phylum in the DSS group, accounting for 53.0%, while *Bacteroidetes* were most abundant in the control and DSS + 2′-FL groups, with proportions of 42.88 and 58.55%, respectively (Figures 5D,E, Supplementary Figure S1). The F/B ratio in the DSS group was significantly greater than that in the control and DSS + 2′-FL groups (*p* < 0.01, Supplementary Figure S1B). Specifically, the family *Muribaculaceae* (originally called S24-7), order *Bacteroidales* (and class *Bacteroidia*) displayed a relative richness in the DSS + 2′-FL group (Figures 5D,E), which might mediate the process in which 2′-FL remits colitis. Nevertheless, *Lachnospiraceae NK4A136, Rumincoccaceae_UCG-014*, and *Iiebacterium valens* were relatively overrepresented in the mice with colitis (*p* < 0.05), while *Faecalibaculum rodentium, Bifidobacterium animalis*, and *Bacteroides caecimuris* were relatively enriched in the control and DSS + 2′-FL groups (*p* < 0.05, Figures 5D,E).

### 2′-FL Altered Mucin-Utilizing Bacteria

**Figure 6:**
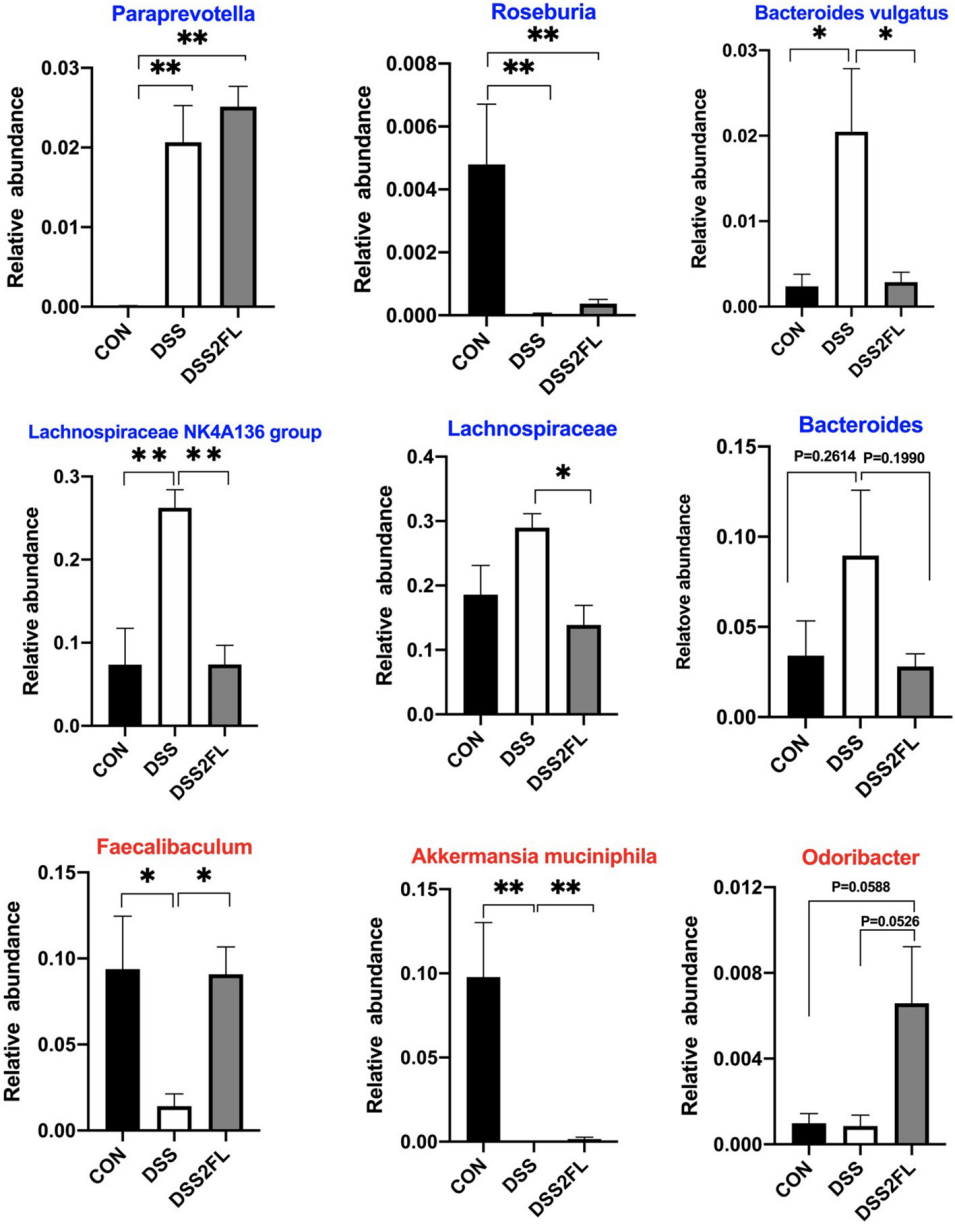
2′-FL alters mucin-utilizing bacteria. Blue font, the bacterial genus encodes cleavage genes and transporters of mucin-associated O-glycans; red font, the bacterial genus encodes cleavage genes of mucin-associated O-glycans. Significance was determined using one-way ANOVA analysis and expressed as mean ± SEM. *p < 0.05; **p < 0.01.

The multilayered mucus covering the colonic surface acts as a major defensive barrier and also serves as “food” for some special bacteria, namely mucin-utilizing bacteria. Comparing colitis mice with or without 2′-FL intake, we found pronounced different abundances on mucin-utilizing bacteria. Specifically, *Paraprevotella, Lachnospiraceae NK4A136, Lachnospiraceae*, and *Bacteroides* were found significantly enriched in the DSS group (*p* < 0.05); however, *Odoribacter, Faecalibaculum*, and *Akkermansia muciniphila* were significantly decreased in the DSS group (*p* < 0.05, Figure 6) compared with the control and DSS + 2′-FL groups.

### 2′-FL Enhanced Mucus Barrier in DSS-Induced Colitis Mice

**Figure 7:**
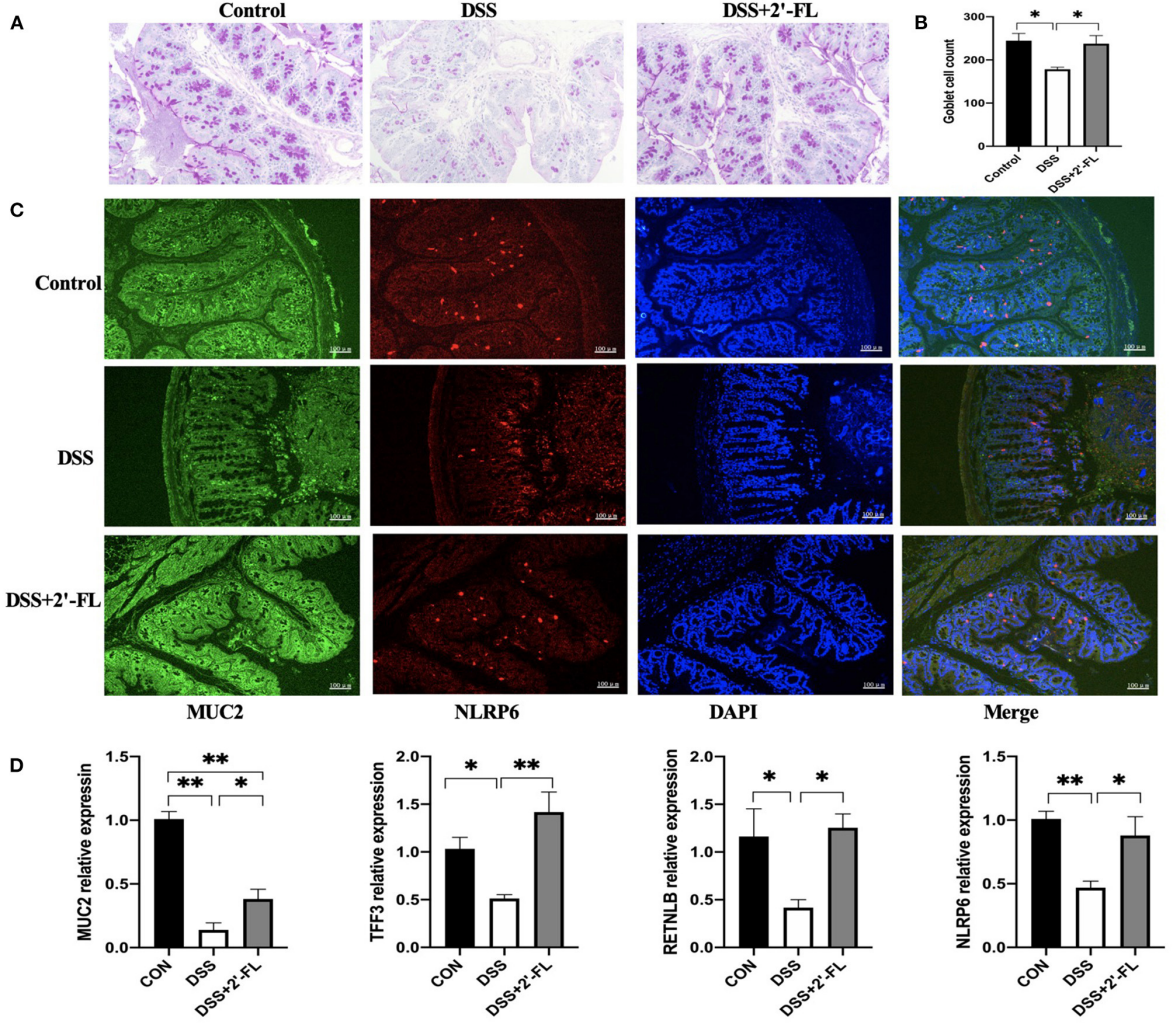
2′-FL increases the number of goblet cells and mucin expression. **(A)** Representative periodic acid-Schiff (PAS)-stained distal colon sections showing goblet cells. Scale bar, 100 μm; **(B)** The number of goblet cells in distal colon; **(C)** Representative fluorescent images of MUC2 (green) and NLRP6 (red) in colon tissue. **(D)** Quantitative RT-PCR showing expression of MUC2, TFF3, RETNLB and NLRP6, relative to GAPDH. Significance was determined using one-way ANOVA and expressed as mean ± SEM. (*n* = 6 for each group). *p < 0.05; **p < 0.01.

Mucin 2 produced by colonic goblet cells is the major component of the colonic mucus barrier. An adequate number of goblet cells is critical in the replenishment of MUC2 mucin. Additionally, TFF3 and RETNLB, the other two proteins secreted by goblet cells, have been shown to be closely related with MUC2 secretion (32). PAS and immunofluorescence staining results showed a significant depletion of goblet cells and MUC2 secretion in the DSS group. Moreover, colonic inflammation resulted in a decrease of TFF3 and RETNLB expression, indicating a dysfunction in goblet cells. After 2′-FL ingestion, the number of goblet cells in colon was increased, so did as MUC2, NLRP6, TFF3, and RETNLB mRNA expressions (Figure 7).

### FMT Validated the Role of Microbiota Regulated by 2′-FL in Protecting Goblet Cells and Enhancing MUC2 Secretion

**Figure 8:**
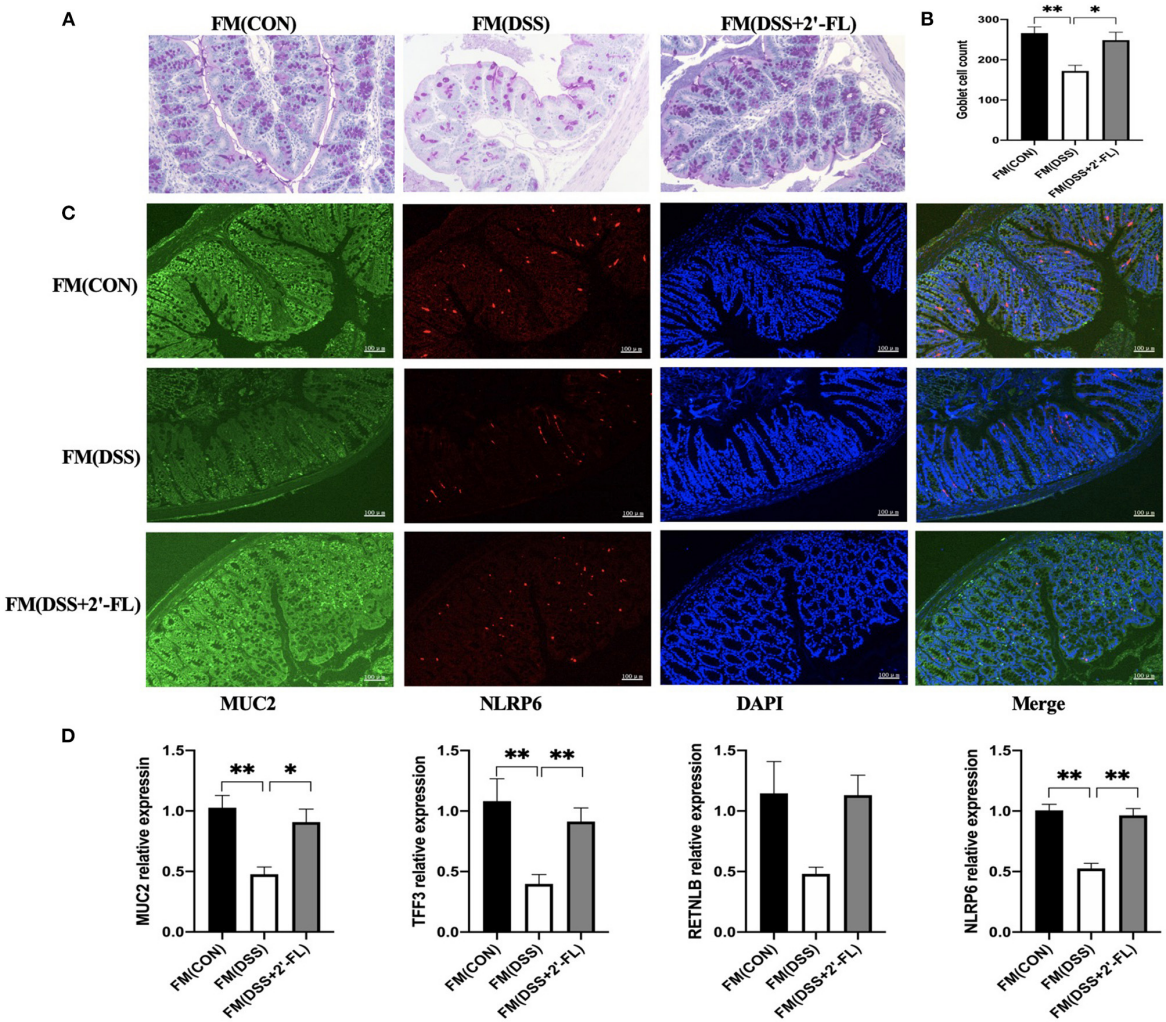
FMT validates the role of microbiota regulated by 2′-FL in protecting goblet cells and enhancing MUC2 secretion. **(A)** Representative periodic acid-Schiff (PAS)-stained distal colon sections showing goblet cells. Scale bar, 100 μm; **(B)** The number of goblet cells in distal colon; **(C)** Representative fluorescent images of MUC2 (green) and NLRP6 (red) in colon tissue. **(D)** Quantitative RT-PCR showing expression of MUC2, TFF3, RETNLB and NLRP6, relative to GAPDH. Significance was determined using one-way ANOVA and expressed as mean ± SEM. (*n* = 6 for each group). *p < 0.05; **p < 0.01.

We further detected the goblet cells and MUC2 secretion in the FM groups and the results showed that after receiving the bacteria, the mice from the DSS + 2′-FL group exhibited a recovery of goblet cells and an increase of MUC2 secretion, as well as TFF3 and RETNLB mRNA expression in colon tissue, even without 2′-FL supplement (Figure 8). Those suggested that gut microbiota reshaped by 2′-FL were involved in the recovery of goblet cell function.

### NLRP6 Is a Negative Regulator for TLR4/MyD88/NF-κB Pathway in Colon Tissue

**Figure 9:**
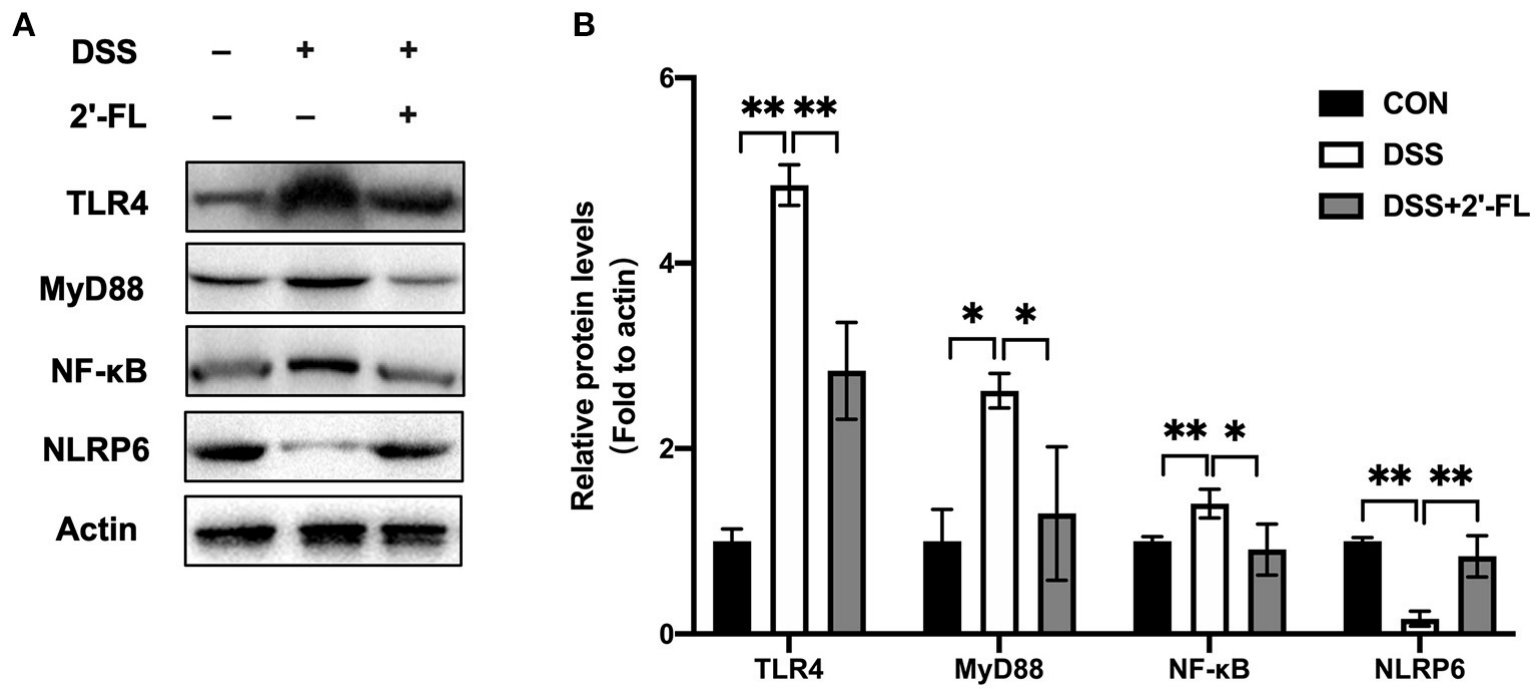
Protein expression of factors in TLR4-related pathway in mouse colon tissue. **(A)** Immunoblot bands of Actin, TLR4, MyD88, NF-κB and NLRP6 in colon tissue. Actin was regarded as an internal reference. **(B)** Densitometric quantitation for normalized proteins relative to Actin (%) in colon tissue, analyzed by Image J software. *p < 0.05; **p < 0.01.

The TLR4-related pathway is well recognized in the inflammation process and plays a role in activating NLRP6, which subsequently promotes goblet cells to secrete MUC2 (15, 16). To further investigate the mechanism of 2′-FL on remitting inflammation, the expression of the TLR4, MyD88, NF-κB, and NLRP6 in colon tissue was detected. The levels of TLR4, MyD88, NF-κB, and TNF-α were significantly upregulated in the DSS group compared with the control (*p* < 0.05), while NLRP6 levels were remarkably downregulated (*p* < 0.05) (Figure 9). The tendency was totally reversed by 2′-FL, as reflected in significantly decreasing levels of TLR4, MyD88, NF-κB, and increasing levels of NLRP6 (*p* < 0.05), indicating that NLRP6 might be a negative regulator for TLR4-related pathway.

## Discussion

Clinical studies have shown that 2′-FL can reduce infant diarrhea, gastrointestinal infection, and the severity of colitis by improving immune functions and inhibiting the colonization of some pathogenic bacteria (33, 34). However, its regulation of the intestinal microbiome in colitis, as well as the potential mechanisms involved therein, has yet to be established. In this study, DSS was used to induce colitis in mice, the pathogenic properties of which is similar to clinical ulcerative colonic disease featured by colonic epithelial injury, endotoxin entry into blood, colonic edema, and excessive inflammation (35), and we further elucidated the protective effect of 2′-FL on this colitis model. Intestinal flora, regarded as an “invisible” organ, has been reported to mediate the function of biochemicals (36). Thus, we hypothesized that intestinal microbiota could affect the protective activity of 2′-FL against colitis. To test this, the feces of donor mice orally treated with DSS+2′-FL were transplanted to recipient mice, which had been excluded from 2′-FL treatment. As expected, after a continuous 7-day FMT and then a successive 7-day DSS treatment, the recipient mice showed a similar protective activity against colitis as the donor mice, despite them not being treated with 2′-FL. It suggested that 2′-FL could remit the colitis in a gut microbiota-dependent manner.

2′-FL cannot be digested by the stomach due to the absence of specific types of galactosidase and, consequently, most of this HMO remain intact until reaching the intestine for the usage of microbes. To further identify the compositional changes in the microbial community, we performed full-length 16S rRNA sequencing. *Firmicutes* and *Bacteroidetes* are the most dominant phyla in the intestine, accounting for approximately 90%, and are considered to contribute to the production of short-chain fatty acids (SCFAs). The level of inflammation-promoting bacterium *Firmicutes* was found to be significantly increased by DSS treatment (37), which was also the case in our experiment, while 2′-FL prevented the induction. The highest F/B ratio in the DSS group, a distinctive sign of obesity-induced inflammation (38, 39) indicated the dysbiosis of the intestinal microbiota. The *Bifidobacterium* genus, enriched in infant intestine, has been reported to be specifically promoted by 2′-FL (12); however, this growth beneficial effect is selective, working only at the presentation of the gene set, such as 1,2-α-L-fucosidase and galacto-N-biose (GNB)/lacto-N-biose I (LNB) in *Bifidobacterium* spp. (40, 41). In this study, *Bifidobacterium animalis*, but not *Bifidobacterium pseudolongum*, was remarkably increased in the DSS + 2′-FL group compared with the DSS group. *Lachnospiraceae NK4A136* group was reported to be positively associated with pathological features of colitis induced by DSS (42, 43). In agreement with previous studies, this study showed that *Lachnospiraceae NK4A136* group was highest in the DSS group and then was significantly decreased by 2′-FL treatment, suggesting that 2′-FL might selectively inhibit some opportunistic bacteria when colitis develops.

The mucus layer is a key component that defends the lamina propria against microbes and pathogens. The fucosidase and sialidase activity of some commensal bacteria utilizing mucins as an energy source likely plays an important role in supporting their colonization and maintaining intestine healthy (44). Family *Muribaculaceae* has been found to be the major mucin monosaccharide foragers, followed by members of *Lachnospiraceae, Rikenellaceae*, and *Bacteroidaceae* families (45). In this study, *Lachnospiraceae* was significantly upregulated by DSS; however, the *Muribaculaceae* were selectively increased by 2′-FL treatment, consistent with study by Li (20). All the results suggested that the composition of mucin-utilization bacteria was remarkably altered by DSS comparing with the control group, while 2′-FL reversed the changes. According to Wlodarska et al., we further analyzed the abundances of mucin-utilizing bacteria containing genes that cleave mucin-associated sugars (cleavage genes) or uptake the cleaved sugars (transporter genes) (46). In this study, *Paraprevptella, Lachnospiraceae NK4A136, Lachnospiraceae*, and *Bacteroides* were found in significantly higher amount in the DSS group; however, *Faecalibaculum, Akkermansia muciniphila*, and *Odoribacter* were significantly decreased in the DSS group. Those alterations in mucin-utilizing bacteria, as well as goblet cells depletion in the DSS group, might lead to an excessive consumption of MUC2, decreased thickness of the mucus layer, and more severe inflammation in the colon. Recovery from those negative alterations, including decreasing the proportion of mucin-utilizing bacteria, increasing goblet cells, and MUC2 secretion, might be the main mechanism by which 2′-FL attenuated the inflammation. Collectively, those results provided a new perspective to understand the relationship between gut microbiota and 2′-FL.

NLRP6, a new member of nod-like receptor family, forms NLRP6 inflammasome and is highly expressed in the intestinal tract and liver (47). In the intestine, NLRP6 regulates IL-18 production, MUC2 expression in goblet cells, and bacterial homeostasis (15, 48). Several studies have shown that the flora signal and metabolic signal, such as taurine, unsaturated fatty acid, histamine and spermine, act as the priming signal to regulate the expression of NLRP6 in intestinal epithelial cells, and the flora component, such as family *Prevotellaceae* and *TM7*, act as the secondary signal to bind NLRP6 to induce the assembly and activation of inflammasome (47, 49, 50). Besides that, TLR ligand activates MyD88-reactive oxygen species (ROS) pathway to activate NLRP6 inflammasome. In this study, after 2′-FL treatment, the family *Muribaculaceae* was significantly changed. All those results encouraged us to test NLRP6 expression in the colon tissue of DSS-induced mice. The results showed that 2′-FL could mitigate the decrease of NLRP6 and MUC2 induced by DSS. We further investigated the roles of 2′-FL in regulating TLR4-related pathway through NLRP6 and we found that 2′-FL treatment downregulated the levels of TLR4, MyD88, and NF-κB and upregulated NLRP6 level, indicating that NLRP6 might be a negative regulator for TLR4-related pathway.

## Conclusion

The findings of this study indicate that 2′-FL ameliorates colitis in a gut microbiota-mediated manner. The underlying protective mechanism is associated with the recovery of goblet cells number and improved MUC2 secretion through regulating mucin-utilizing bacteria. Besides, 2′-FL exerts anti-inflammatory effects by targeting the TLR4/MyD88/NF-κB-related inflammation pathway through the upregulation of NLRP6 expression. Taken together, 2′-FL might be a valuable candidate for the development of functional foods to protect from intestinal inflammation.

## Data Availability Statement

The data presented in the study are deposited in the NCBI repository, accession number: BioProject PRJNA794307.

## Ethics Statement

The animal study was reviewed and approved by Committee on the Ethics of Animal Experiments of the Chinese Academy of Agricultural Sciences (Beijing, China; permission number: IAS-2021-03).

## Author Contributions

JW and HL conceived the original idea. NZ, CB, and VD designed the experiments. QY and LF performed the experiments and analyzed the data. QY wrote the manuscript. All authors contributed to the article and approved the submitted version.

## Funding

This study was supported by grants from the Scientific Research Project for Major Achievements of Agricultural Science and Technology Innovation Program (CAAS-ZDXT2019004), the Ministry of Modern Agro-Industry Technology Research System of China (CARS-36), and the Agricultural Science and Technology Innovation Program (ASTIP-IAS12).

## Conflict of Interest

The authors declare that the research was conducted in the absence of any commercial or financial relationships that could be construed as a potential conflict of interest.

## Publisher's Note

All claims expressed in this article are solely those of the authors and do not necessarily represent those of their affiliated organizations, or those of the publisher, the editors and the reviewers. Any product that may be evaluated in this article, or claim that may be made by its manufacturer, is not guaranteed or endorsed by the publisher.

## Supplementary Material

Figure S1 Effects of 2′-FL on composition of gut microbiota in C57BL/6J mice. A, Stacked bar plots of species distribution at phylum, family, species levels in each sample. B, The changes of *Firmicutes, Bacteroidetes, Firmicutes/Bacteroidetes* (F/B) and *Actinobacteria* in three groups. Significance determined using one-way ANOVA analysis and expressed as mean ± SEM. ^*^*P* < 0.05. ^**^*P* < 0.01.
