# Supplementary figures and images for: 2′-Fucosyllactose Ameliorates Inflammatory Bowel Disease by Modulating Gut Microbiota and Promoting MUC2 Expression
Source: Front Nutr. 2022 Feb 17;9:822020. doi: 10.3389/fnut.2022.822020 (PMC8892212; doi:10.3389/fnut.2022.822020)

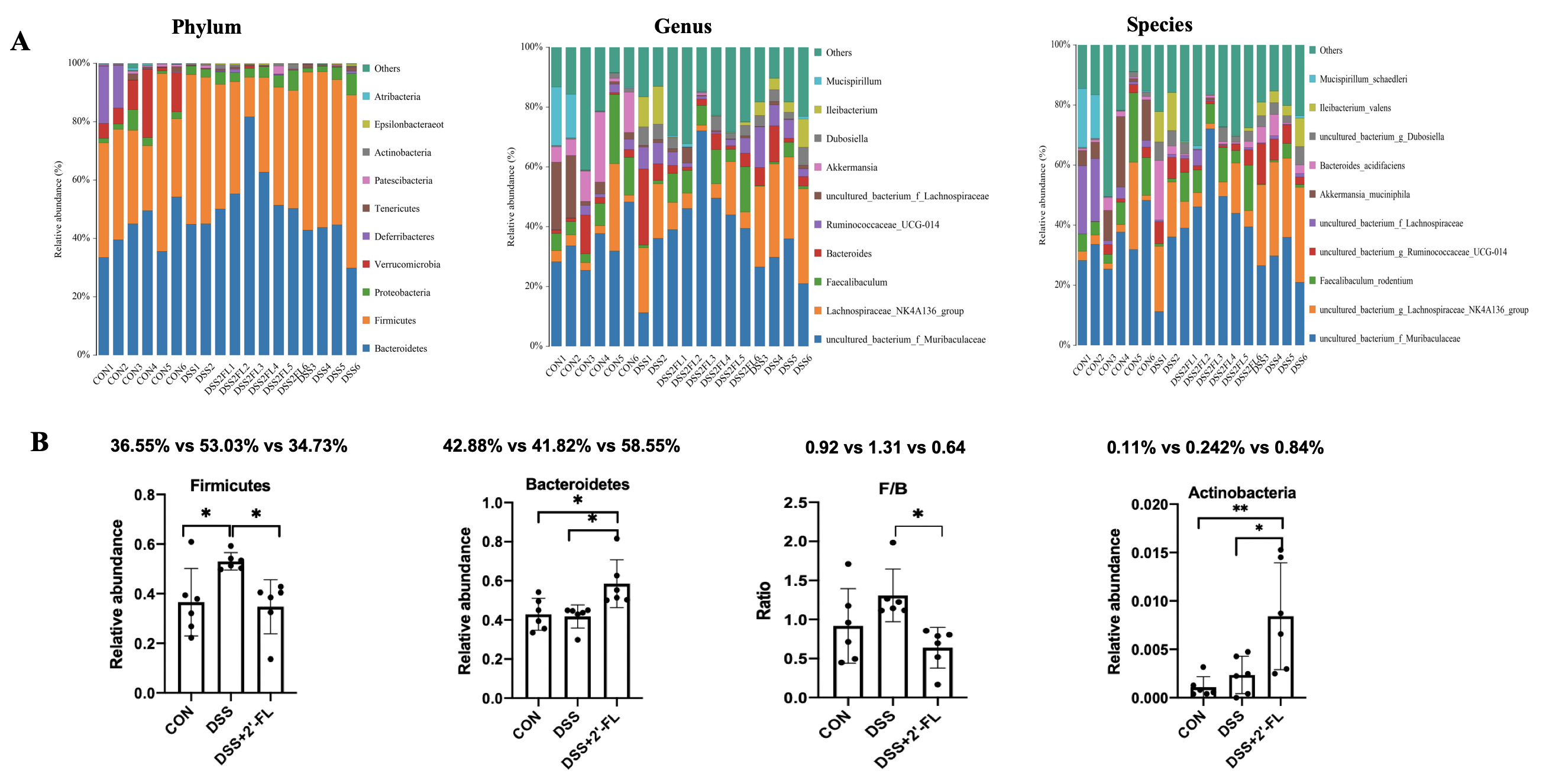

Supplement: Figure S1 — Effects of 2′-FL on composition of gut microbiota in C57BL/6J mice. A, Stacked bar plots of species distribution at phylum, family, species levels in each sample. B, The changes of Firmicutes, Bacteroidetes, Firmicutes/Bacteroidetes (F/B) and Actinobacteria in three groups. Significance determined using one-way ANOVA analysis and expressed as mean ± SEM. *P < 0.05. **P < 0.01. [file Image_1.png]
